# Identifying High-Risk Chronic Lymphocytic Leukemia: A Pathogenesis-Oriented Appraisal of Prognostic and Predictive Factors in Patients Treated with Chemotherapy with or without Immunotherapy

**DOI:** 10.4084/MJHID.2016.047

**Published:** 2016-10-15

**Authors:** Sara Martinelli, Antonio Cuneo, Luca Formigaro, Maurizio Cavallari, Enrico Lista, Francesca Maria Quaglia, Maria Ciccone, Antonella Bardi, Eleonora Volta, Elisa Tammiso, Elena Saccenti, Olga Sofritti, Giulia Daghia, Massimo Negrini, Melissa Dabusti, Paolo Tomasi, Sabrina Moretti, Francesco Cavazzini, Gian Matteo Rigolin

**Affiliations:** Section of Hematology, Azienda Ospedaliero-Universitaria S.Anna, Ferrara, Italy

## Abstract

Chronic lymphocytic leukemia (CLL) displays an extremely variable clinical behaviour. Accurate prognostication and prediction of response to treatment are important in an era of effective first-line regimens and novel molecules for high risk patients. Because a plethora of prognostic biomarkers were identified, but few of them were validated by multivariable analysis in comprehensive prospective studies, we applied in this survey stringent criteria to select papers from the literature in order to identify the most reproducible prognostic/predictive markers. Each biomarker was analysed in terms of reproducibility across the different studies with respect to its impact on time to first treatment (TTFT), progression free survival (PFS), overall survival (OS) and response to treatment. We were able to identify the following biomarkers as the most reliable in guiding risk stratification in the daily clinical practice: 17p-/*TP53* mutations, *IGHV* unmutated configuration, short telomeres and 11q-. However, the method for measuring telomere length was not validated yet and 11q- was predictive of inferior OS only in those patients who did not receive FCR-like combinations. Stage and lymphocytosis were predictive of shorter TTFT and age, high serum thymidine kinase levels and poor performance status were predictive of shorter OS. Using our criteria no parameter was found to independently predict for inferior response to treatment.

## Introduction

**Figure 1:**
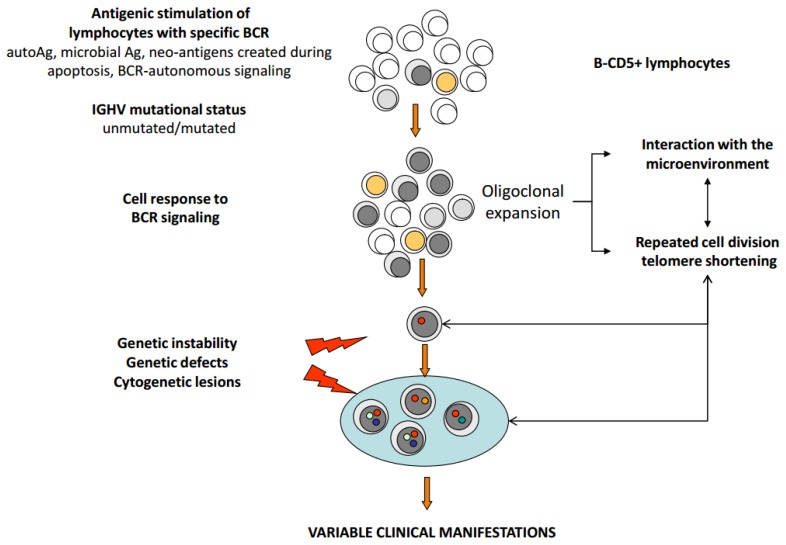
Pathogenesis of CLL. Autonomous BCR signalling and BCR stimulation by autoantigens, external antigens or by neo-epitopes generated during the apoptotic process cause variable cell responses (cell activation or anergy), depending on the *IGHV* mutational status. Oligoclonal expansion, cell divisions and telomere shortening occur, within a complex interaction with the microenvironment. Primary and secondary molecular-cytogenetic lesions determine clonal expansion and tumor progression, resulting into a heterogeneous clinical behaviour.

**Figure 2:**
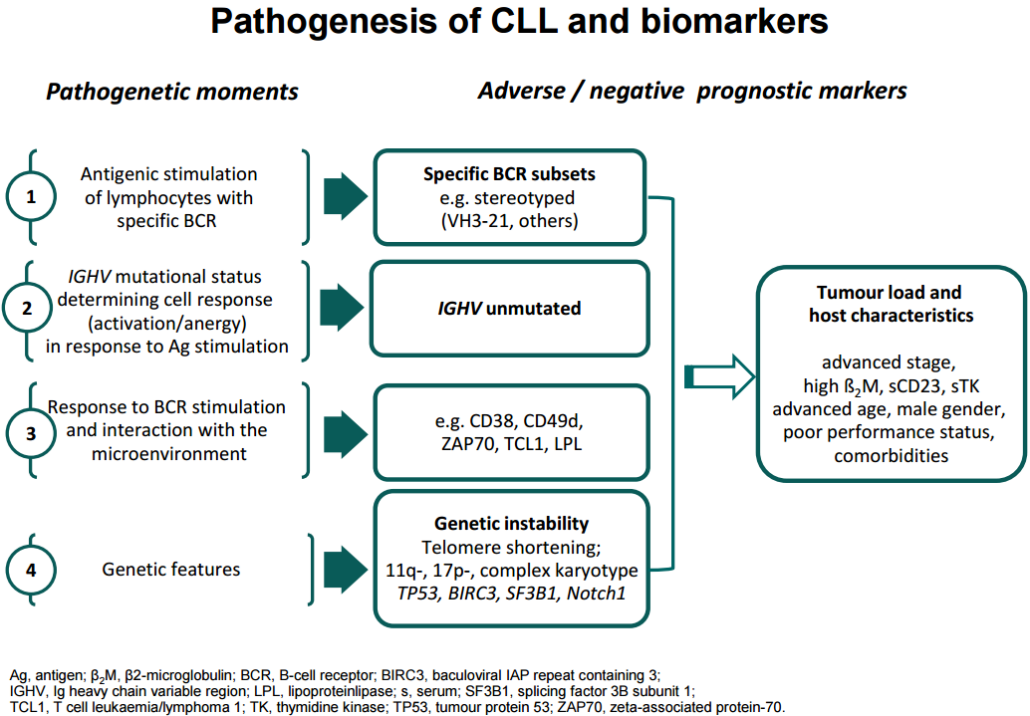
Pathogenic steps and corresponding prognostic markers.

Chronic lymphocytic leukemia (CLL) displays a variable clinical behaviour, with many patients living for years without symptoms and other patients requiring early therapeutic intervention attaining short lasting responses and succumbing to their disease in a few years. Therefore, survival in this chronic lymphoproliferative disorder largely depends on the rapidity of disease progression and on the quality and duration of response to treatment. The availability of effective first-line regimens^1^–^6^ makes prognostication and prediction of response to treatment an important exercise in clinical practice, especially in young and/or fit patients who may benefit of aggressive regimens including allogeneic bone marrow transplantation.^7^,^8^ Clinical staging is a simple measure of disease burden and still represents a convenient, yet insufficient means of assessing prognosis, because it does not identify those patients with limited disease who have a high probability to progress, and it does not predict the quality and duration of response to treatment. A plethora of biomarkers have been identified in the last decades which may predict disease outcome,^9^ but few of them were validated in the context of prospective studies using adequate statistic considerations to weigh the risk of each parameter by multivariable analysis. Meanwhile, our understanding of CLL biology greatly improved providing a basis for a better understanding of the biologic role of prognostic markers.^10^ The pathogenesis of CLL is the result of a complex interplay between i) lymphocytes carrying a restricted repertoire of BCR,^11^ ii) the mutational status of the variable portion of the immunoglobulin heavy chain (*IGHV*) gene determining different behaviour of neoplastic lymphocyte in response to antigen stimulation, iii) cell activation and interaction with the microenvironment,^12^ iv) genetic lesions^13^ (Figure 1). Each of these fundamental mechanisms is associated with specific biomarkers, that identify high-risk CLL, as summarized in Figure 2. Central to this categorization of prognostic markers is the concept that antigenic stimulation of neoplastic lymphocytes with a restricted set of BCR may promote inhibition of apoptosis, survival and proliferation within the lymph node and bone marrow microenvironment, with consequent multiple cycles of cell division, telomere shortening and genetic instability, with disease-host interactions ultimately shaping variable clinical phenotypes (Figure 2).^14^

In this review clinicobiologic features predicting outcome are discussed in correlation with their pathogenic role and applicability in clinical practice.

## Eligibility Criteria and Literature Search

Based on previous analyses that identified clinical and biologic characteristics having prognostic significance,^9^,^10^,^15^–^17^ the following 18 biomarkers were included in this survey: stereotyped receptors and BCR subsets, CD38, ZAP70, CD49d, *IGHV* gene mutational status, 17p-/*TP53* mutations, 11q- telomere length, complex karyotype, *NOTCH1* and *SF3B1* mutations, age, gender, performance status, stage, lymphocytosis, beta-2-microglobulin, thymidine kinase.

A literature search was then performed to identify studies on the prognostic value of these biomarkers in CLL. We searched PubMed to identify all citations from January 2000 to April 2016 describing the role of the selected parameters in predicting the outcome for newly diagnosed CLL patients. The search was performed using a combination of MeSH controlled vocabulary and text words. The following terms were used: “Leukemia, Lymphocytic, Chronic, B-Cell”[Mesh], “Prognosis”[Mesh], “Clinical Trial” [Publication Type], “Receptors, Antigen, B-Cell”[Mesh], CD38, ZAP70, CD49d, IGHV, IGVH, 17p[All Fields], TP53[All Fields], 11q[All Fields], “Telomere”[Mesh], telomere, complex karyotype, NOTCH1, SF3B1, “beta 2-Microglobulin”[Mesh], thymidine kinase.

Only full length publications satisfying the following requirements were included in the review: i) English language; ii) at least 100 patients included; iii) multivariate analysis including salient clinical data and genetic testing (*IGHV* mutational status, 17p deletion and 11q deletion); iv) prospective design of the study (clinical trial) or single/multicentre study using a learning cohort and a validation cohort or consecutive series; v) at least one endpoint being time to first treatment (TTFT), progression free survival (PFS), overall survival (OS), overall response rate (ORR) or complete response (CR) rate. Manuscripts describing the prognostic impact of the selected parameters in the context of patients starting unconventional or experimental treatment were not included, as well as studies including patients with monoclonal B-cell lymphocytosis.

The search criteria identified 3,845 citations. After duplicate removal and evaluation of all remaining manuscripts, 27 papers met the criteria for inclusion in this study. The characteristics and salient data of these papers are presented in Table 1.

## Results

### Predictors of outcome (TTFT, PFS and OS)

**Figure 3:**
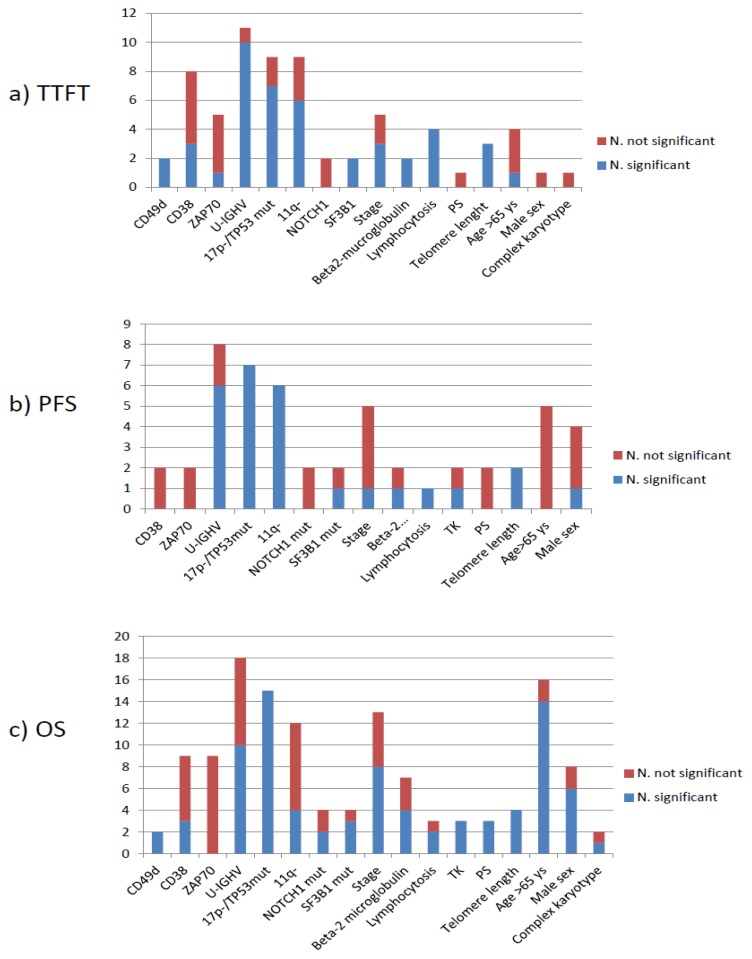
Number of studies assessing the impact of each parameter in terms of a) TTFT; b) PFS; c) OS: the blue part of each column represents the number of studies showing independent negative impact on prognosis (“significant”); the red part represents the number of studies showing no prognostic impact (“not significant”).

Figure 3 represents, for each parameter, the total number of studies analyzing its prognostic impact and the number of studies in which the parameter showed independent prognostic significance in terms of TTFT, PFS and/or OS. Hazard ratios for each marker are reported in Table 1.

#### 1) BCR repertoire and stereotyped receptors

##### Pathogenic role

CLL lymphocytes express a restricted set of BCR due to non-random usage of gene families coding for the variable portion of the Ig.^11^ Furthermore, some CLL cases express highly homogeneous sequences of the heavy chain complementarity determining region 3 (HCDR3), a phenomenon referred to as “stereotyped” BCR^18^ that was shown to occur in up to one-third of the cases.^19^–^21^

The similarity of the BCR from various CLL patients suggests that the precursors of B-CLL cells were chosen for their antigen-binding capabilities by antigen(s) of restricted nature and structure.

##### Prognostic impact

Biased BCR usage and stereotyped receptors did not meet the criteria adopted in this review. The prognostic significance of stereotyped BCR was only assessed in studies that did not include a comprehensive assessment of salient genetic parameters, and no prospective clinical trial was designed including determination of BCR stereotypy in the diagnostic workup. CLL with stereotyped BCR showed shorter TTFT in a study including genetic parameters;^22^ however no multivariable analysis was performed in this analysis.

#### 2) Interaction with the microenvironment and activation markers

##### Pathogenic role

The interaction between neoplastic lymphocytes responding to BCR stimulation and the microenvironment plays a fundamental role in CLL pathogenesis.^23^ As a consequence, the natural history of the disease is dictated, in part, by lymphocyte survival and growth in the lymph nodes that favours the accumulation of genomic alterations, especially within proliferation centres and/or CD38-positive cells.^24^,^25^ Biomarkers reflecting the capability to respond to BCR engagement or to adhere to niches in the bone marrow or lymph node (i.e. CD38, ZAP70, CD49d, lipoprotein lipase, TCL1 expression) were correlated with progressive disease^26^–^30^ and, to the contrary, a signature of anergic lymphocyte that poorly responds to BCR stimulation was observed in indolent CLL.^31^,^32^

##### Prognostic impact

The threshold for CD38-positivity varied across studies. In a comprehensive analysis of 1154 early stage CLL seen at 4 European centres,^33^ CD38+ was predictive of a shorter TTFT (median 9.3 years vs not reached) and shorter survival (median 14,7 years) by multivariable analysis (Table 1). Interestingly a more rapid disease progression requiring treatment was observed in CD38 positive CLL in patients with “favourable” genetic profile.^25^ However, the prognostic significance of CD38 was overcome by more robust genetic parameters in several clinical trials, as shown in Figure 3.^34^–^36^

ZAP-70 was predictive of a shorter TTFT in only one study,^37^ and no impact was shown on PFS or OS in all studies using a complete assessment of prognostic parameters when the multivariable analysis was applied.^35^,^38^–^43^ One study showed an independent impact of ZAP70 on OS;^44^ however, it was not considered in this survey as it used an unusually low expression level (>10%) as a positivity cut-off and because the detection method is not reproducible.^9^

CD49d expression was predictive of shorter interval between diagnosis and disease progression, with a median TTFT of approximately 4 years in CD49d-positive cases.^38^,^44^ Its predictive value was confirmed by multivariable analysis in two analyses^37^,^41^ and an independent adverse impact on OS was documented in two studies using heterogeneous treatment.^37^,^39^

No study with comprehensive assessment of prognostic markers was performed to assess the independent prognostic impact of lipoproteine lipase and TCL1 overexpression.

#### 3) *IGHV* mutational status and genetic features

##### Pathogenic role

Neoplastic lymphocytes carrying ≤2% mutations of the *IGHV* gene compared to the nearest germline sequence are referred to as “unmutated” CLL. The lymphocytes in this CLL subset respond to antigen stimulation by activating intracellular signalling and entering the G1 phase of the cell cycle more efficiently than *IGHV* “mutated” CLL.^23^,^45^,^46^ Consequently neoplastic lymphocyte carrying unmutated *IGHV* sequence, i) undergo more cell divisions in vivo as shown by incorporation of deuterated water^12^ and, ii) carry shorter telomeres and accumulate more genomic defects^47^ than lymphocytes with mutated *IGHV*.

The molecular pathogenesis of CLL is centred around some lesions. i.e. TCL1 or miR-29 overexpression and/or miR15a-16 deletion, producing the disease in the animal model.^48^–^50^ These lesions are associated with a number of chromosomes and genetic aberrations which may appear soon during clonal expansion (13q-, +12, *MYD88*, *NOTCH1* mutations), or later following disease progression or selection by treatment (11q-, 17p-, mutations of *TP53, ATM, SF3B1, BIRC3*).^51^,^52^ Disruption of the *TP53* and *ATM* pathway is associated with resistance to DNA damaging agents and genetic instability leading to the emergence of subclones accounting for disease progression.^9^

##### Prognostic impact

###### Unmutated *IGHV* sequences

Ever since the first reports,^26^,^53^ unmutated *IGHV* proved to be a robust unfavourable prognostic marker.^16^ In a prospective study by Shanafelt and coworkers^38^ TTFT in 1004 CLL was 2,8 years in unmutated CLL as compared to 11 years in mutated CLL. In our analysis, a significantly shorter TTFT with very high hazard ratios (Table 1) was observed in 8 prospective series, 4 of which had enrolled more than 700 cases. PFS was shorter in *IGHV* unmutated CLL in 2 large studies using fludarabine and cyclofosfamide (FC) or fludarabine, cyclofosfamide and rituximab (FCR)^2^ or chlorambucil (Chlor), fludarabine (F) or FC^36^ and the observed difference held when including assessment of new gene mutations (i.e. *SF3B1* and *NOTCH1*) and telomere length in the multivariable model.^54^–^56^ In the CLL4 trial of the GCLLSG using F or FC the *IGHV* configuration in 294 patients showed no independent impact on PFS^57^ as was the case with a US Intergroup Phase III Trial E2997 that assessed the *IGHV* mutational status in 195 patients;^35^ however these 2 analyses were numerically smaller than the other studies. Interestingly, a shorter survival was noted in *IGHV* unmutated CLL in a number of large studies (Table 1, Figure 3) with 85,1% five-year OS in unmutated CLL vs 91.4% five-year OS in mutated CLL in a recent analysis that pooled data form 3 randomized studies of the GCLLSG.^34^

###### Telomeres

Telomeres are specific non-coding nucleotide sequences consisting of 6–12 kbp of TTAGGG-repeats located at the ends of eukaryotic chromosomes that are necessary for the complete replication and stability of the chromosome. Because they are eroded upon each cell division, their length reflects the replicative history of a cell.^58^ In CLL cells telomeres are shorter that in normal B-lymphocytes^59^ and those patients with telomere length below the median observed value were found to frequently carry unmutated *IGHV* gene^60^ and unfavourable prognosis.^61^ In our analysis, a significantly shorter TTFT was found in CLL with shortened telomeres in 3 studies,^41^,^62^,^63^ 2 of which had partially overlapping cohorts (Table 1). Short telomeres were independently associated with inferior PFS in two large studies assessing the most significant prognostic parameters^43^,^56^ and negatively impacted on OS in 4 studies that included patients treated with various regimens(Table 1, Figure 3). 43,56,62,63

###### 17p-/*TP53* mutations and 11q deletion

These aberrations alter cell-cycle and DNA-repair pathways. In the case of a functional *TP53* pathway, DNA damage activates p53 through the activation of ATM, thus inducing cell cycle arrest through p21 and apoptosis. 17p13 deletion causes loss of one *TP53* allele and determines resistance to DNA-damaging drugs through haploinsufficiency.^64^ 17p13 deletion is associated in the vast majority of cases with inactivating mutations of the remaining *TP53* allele. *The tp53* mutation may occur independent of 17p deletion and may inactivate p53 function by a dominant negative effect or by duplication of the chromosome segment containing the mutated *TP53* gene, a genetic rearrangement referred to as uniparental disomy.^65^ Disruption of the *TP53* pathway by deletion and/or mutation leads to resistance to apoptosis and genetic instability and indeed 17p- patients frequently exhibit complex chromosome defects and multiple genetic lesions.^55^,^66^ The negative impact of 17p13 deletion on OS and clinical response to fludarabine were clearly documented as early as 1995,^67^ and a similar negative impact was shown to be associated with *TP53* mutations, independent of the presence of 17p13 deletion.^36^,^57^,^68^ Interestingly, minor clones carrying *TP53* mutations (<20% of the cells) were equally shown by sensitive next generation sequencing techniques to predict for an inferior outcome.^69^ The papers selected for this review show that the aberrations leading to disruption of the *TP53* pathway, either by 17p deletion or by inactivating *TP53* gene mutation, or both, have a deleterious impact on all outcome measure, as shown in Table 1 and Figure 3. A hazard ratio of 3,96 of being treated after 26 months vs. patients with 13q- as single aberration was recorded in a large prospective study of 930 patients^70^ and more rapid disease progression requiring treatment was observed in one large multicentre study^71^ and in one single centre study.^72^ At variance, one large analysis conducted at a referral laboratory receiving samples from several institutions did not find an impact on TTFT for *TP53* mutations.^42^ Even though the presence of mutated *IGHV* gene and early stage reduced the adverse effect of this genetic aberration in a minority of patients,^73^,^74^ 17p-/*TP53* mutations definitely identify patients with dismal outcome with the current treatment regimens using alkylating agents, purine analogues, and anti CD20 monoclonal antibodies. Indeed shorter PFS and OS were uniformly reported in all the studies (Table 1, Figure 3). Interestingly, *TP53* disruption appears to be associated with more frequent progression in the relapsed/refractory setting under novel active BCR-targeted therapies.^75^–^77^

11q22–23 deletion characterizes a CLL subtype with extensive nodal involvement and inferior prognosis.^78^ The minimal region of deletion involves the ataxia-teleangiectasia mutated *(ATM*) gene, with concurrent mutation of the remaining *ATM* allele occurring in 30–40% of 11q- cases and causing extreme telomere shortening^79^ and dismal prognosis.^80^ Because ATM is a very large gene, mutational studies were not performed in clinical trials and papers included in this review refer to patients with 11q-. TTFT and PFS were shorter in patients with 11q- in the majority of studies (Table 1, Figure 3). It is noteworthy, however, that the negative impact on PFS was overcome by adding rituximab to FC.^2^ Likewise, some reports demonstrated an adverse impact of 11q- on OS,^81^ whereas more studies using effective regimens based on purine analogues and alkylating agents with rituximab^2^,^55^,^82^ did not detect any difference by multivariable analysis. Thus, it appears that the negative prognostic impact of 11q- is abated in young and/or fit patients treated by modern chemoimmunotherapy regimens.

###### Lesions involving *NOTCH1* and *SF3B1*

Mutations causing activation of the *NOTCH1* pathway, with consequent activation of non-canonical *NFkB* signalling, may promote cell survival and resistance to apoptosis.^83^–^85^ *SF3B1* mutations cause altered splicing of a number of targets including FOXP1, that encodes for a forkhead transcription machinery,^86^ promoting resistance to fludarabine-based treatment through as yet unknown mechanisms.^87^ Shorter TTFT was noted in patients with *SF3B1* mutations in 2 studies,^42^,^88^ whereas *NOTCH1* mutation did not predict for more rapid disease evolution (Table 1). Likewise, *NOTCH1* mutations did not impact on PFS, whereas shorter PFS was observed in *SF3B1* mutated patients treated by FC or FCR in the CLL8 study.^55^ This correlation was not observed in the UKCLL4 study using chlorambucil, or fludarabine with or without cyclophosphamide (Table 1). 54 Rossi et al. showed an adverse impact on OS of NOTCH1 mutation.^89^ OS was shorter in *SF3B1* and *NOTCH1* mutated patients in the UKCLL4 trial^54^ and not in the CLL8 trial (Table 1).^55^ It is noteworthy that two more studies showed an independent impact of the *SF3B1* mutation on overall survival (Figure 3).^42^,^90^

###### Conventional banding analysis

Metaphase karyotyping represented the first biomarker having prognostic significance in CLL, in the seminal paper by Juliusson et al..^91^ More recently complex karyotype was included in a prognostic scoring system predictive of time to first treatment and overall survival.^92^ The presence of chromosome aberrations was predictive of an inferior outcome in those patients without detectable aberrations by fluorescence in situ hybridization (FISH).^93^ Unbalanced chromosome translocations, frequently occurring in the context of complex karyotype, were shown to be independent prognostic factors in a study^94^ and complex karyotype predicted for a shorter TTFT^71^ and OS^95^ by multivariable analysis. The prognostic value of complex karyotype was also demonstrated in relapsed/refractory CLL patients treated with ibrutinib-based regimens, where this parameter showed a stronger impact on the outcome than del(17p).^96^ After data for this review had been collected, a comprehensive prospective study on 161 CLL patients with relevant comorbidity showed the independent prognostic role of complex karyotype for survival following chlorambucil-based chemoimmunotherapy.^97^ Thus, there is mounting evidence that chromosome banding analysis with novel efficient mitogens may substantially contribute to the identification of CLL patients with adverse prognosis.

#### 4) Disease/ host characteristics

##### Pathogenic role

Markers of tumor burden and proliferative activity of neoplastic cells may have an obvious impact on prognosis. Clinical stage, peripheral lymphocytosis, bone marrow infiltration pattern, serum markers such as soluble CD23 and β2-microglobulin, an extracellular protein associated with the class I major histocompatibility complex, represented for many years valuable prognostic markers.^98^ Likewise, markers of proliferative activity, i.e. lymphocyte doubling time and serum thymidine kinase-1 (TK1), a cellular enzyme involved in the DNA synthesis in the G1/S phase of the cell cycle and reflecting the number of dividing neoplastic cells, were shown to have a significant prognostic impact.

Age and gender may have an evident impact on prognosis. According to the global health observatory data repository of the WHO (available at the link http://www.who.int/gho/mortality_burden_disease/life_tables/situation_trends/en/) life expectancy in 2012 at the age of 60 was 19 and 23 years in Europe for males and females respectively (21 and 24 years in the Americas). Given the availability of treatments that are able to modify the natural history of the disease and to prolong survival,^99^ host characteristics such as performance status and comorbidities may also have a significant role as prognosticators.^34^,^55^

##### Prognostic impact

The standardization of chemiluminescence immunoassay for the assessment of serum thymidine kinase-1 (TK) levels significantly facilitated the introduction of this marker into clinical practice,^100^ and age-related normal reference values were recently defined and validated.^101^ When this test was included among diagnostic workup within prospective trials, raised TK levels (i.e. ≥10 U/L) were predictive of shorter PFS^55^ and survival in the GCLLSG trials.^34^ Likewise, serum beta-2-microglobulin levels proved an independent prognostic parameter on more outcome measures in several studies (Table 1, Figure 3).

Lymphocytosis, ECOG performance status, stage and gender showed a variable impact on prognosis. Direct quantitation of the disease burden such lymphocytosis and stage are predictive of shorter TTFT and have a variable impact on other outcome measures,^102^ however, they lacked significant prognostic value in the largest analysis based on pooled data from several GCLLSG studies.^34^

Age and PS had no impact on TTFT and PFS, whereas they showed significant impact on OS (Table 1, Figure 3).

### Factors predictive of response to treatment

No paper with the stringent characteristics adopted in our analysis was found to address the impact of biomarkers on predicting response treatment, with two notable exceptions:

#### a) 17p-/TP53 mutations and 11q-

There is evidence that carrying 17p-/*TP53* mutation conveys a low probability to achieve a clinical response with chemoimmunotherapy.^67^ ORR was lower in 17p-/*TP53* mutated patients in 3 large randomized trials comparing FC vs FCR,^2^ F vs FC^57^ and Chlorambucil vs F vs FC.^81^ Even though these data were not validated by multivariable analysis, the probability to attain a meaningful response was very low for the 17p- group in the CLL8 trial, with a CR rate of 0% and 5% and an ORR rate of 51,6% and 75% with FC and FCR, respectively.^2^,^55^ Interestingly, *TP53* mutations were shown to represent an independent predictive factor of shorter time to chemorefractoriness.^68^

#### b) Lack of efficacy of anti CD20 monoclonal antibodies rituximab and ofatumumab in NOTCH1 mutated patients

Recent evidence was provided that response to chemoimmunotherapy containing anti-CD20 monoclonal antibodies may be negatively influenced by the presence of *NOTCH1* mutations. The adjunct of ofatumumab to chlorambucil significantly improved PFS in the total population of a phase III study;^103^ while not influencing response to treatment, *NOTCH1* mutation was associated with shorter PFS in the ofatumamuab plus chlorambucil arm (17.7 months vs. 23.3, HR 1.86 p=0.01), making the addition of the monoclonal antibody to chlorambucil irrelevant in terms of PFS over chlorambucil alone.^103^ In the CLL8 study^55^ there was no significant difference in terms of ORR in the FC arm and in the FCR arm depending on the *NOTCH1* mutation status; however, while the association of rituximab with FC improved ORR in patients with wild type *NOTCH1* (88,1 vs. 96,6%; p<0.01), no difference in ORR was noted in *NOTCH1* mutated patients in the FC and FCR arms (87,1% vs. 90%). PFS was superimposable in *NOTCH1* mutated patients in the FCR (34,2 months) and FC arm (33,9 months). Taken together these data suggest that *NOTCH1* mutation is a predictive marker for reduced benefit from the addition of rituximab or ofatumumab to chemotherapy. Interestingly, CLL cases carrying *NOTCH1* mutations are characterized by low CD20 expression levels deriving at least in part from histone deacetylase-dependent transcriptional repression, an observation that may explain the low sensitivity of these patients to anti-CD20 monoclonal antibodies.^104^

## Conclusions

**Figure 4:**
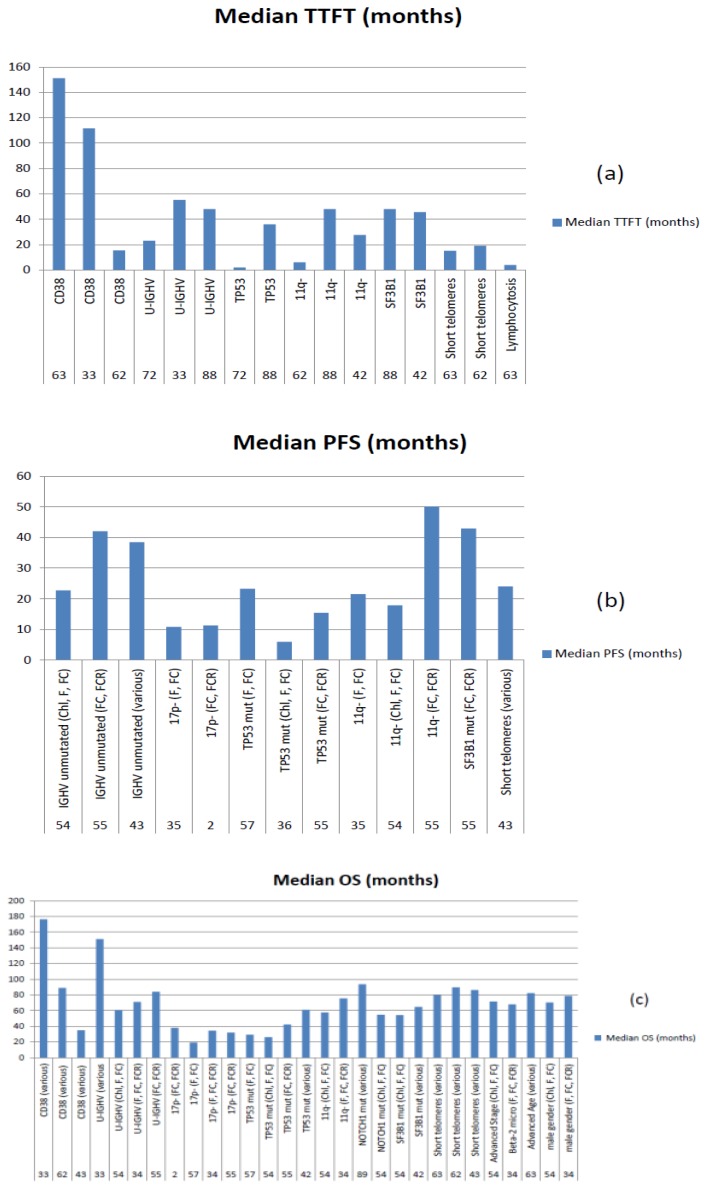
Time to first treatment (a), PFS (b) and OS (c) in the presence of the unfavourable biomarker (reference number at the bottom of each column). Chl: chlorambucil; F: fludarabine; FC: fludarabine and cyclophosphamide, FCR for FC plus rituximab.

Using stringent criteria we were able to identify 16 parameters, i.e. CD38, CD49d, unmutated *IGHV*, 17p-/*TP53* mutations, 11q-, telomere length, complex karyotype, *NOTCH1* and *SF3B1* mutations, age, gender, performance status (PS), stage, lymphocytosis, beta-2-microglobulin, thymidine kinase, with unfavourable prognostic significance on TTFT, PFS and/or OS by multivariable analysis in prospective clinical trials or in the context of well-organized studies. The observed TTFT, PFS, and OS for each of these markers in the corresponding studies is shown in Figure 4.

Since 17p-/*TP53* mutations, unmutated *IGHV* gene configuration and 11q- proved to be independent predictors of outcome in at least 2/3 studies (Figure 3) and their detection methods were standardized,^105^–^107^ these markers are to be considered the most reliable for usage in clinical practice. Telomere length proved a reproducible predictor of the outcome but the detection method is not standardized yet, even if a recent study confirmed the reproducibility of results obtained with monochrome multiplex Q-PCR (MMQ-PCR) and single telomere length analysis (STELA), opening the way for the assay standardisation.^56^ Complex karyotype as detected by stimulation with novel mitogens^66^ may represent a novel predictor of unfavourable outcome.^71^,^95^–^97^ While simple measures of disease burden such as stage and lymphocytosis do not play a role anymore as prognostic markers, host characteristics such as poor PS and advanced age still play a relevant role in predicting OS.

It is noteworthy that evolution of treatment may overcome the significance of some of these prognostic factors; thus, while maintaining its predictive value on OS in patients not eligible to modern chemoimmunotherapy regimens, 11q- lost its unfavourable significance in those patients treated by FCR.^34^,^55^ Importantly, mechanism-based treatment in the refractory/relapsed setting showed high efficacy in “high risk” patients;^75^ thus the introduction of new oral agents targeting kinase signalling or BCL2 will likely change the significance and role of many of these markers.^108^

## Figures and Tables

**Table 1:** Characteristics of the studies showing independent prognostic significance for one or more biomarkers on TTFT, PFS and OS analysis.

Reference	Study (Aviano series/ multicenter; GCLLSG CLL8)	N of patients	Median follow up (months)	Treatment	Unfavourable prognostic significance at multivariable analysis [Hazard ratio]		
					TTFT	PFS	OS
35	Grever, JCO 2007	235	35	F vs FC	NA	17p- [3.43], 11q- [1.90]	NA
39	Gattei, Blood 2008	303	74.4	various	NA	NA	CD49d[3.52], u*IGHV* [6.53] age [6.85]
72	Dicker, Leukemia 2009	193	58.3	various	u*IGHV* [3.44], *TP53* [6.46]	NA	NA
68	Rossi, CCR 2009	308	54.3	various	NA	NA	*TP53* [3.20], age [4.98], stage [3.01]
63	Rossi, Leukemia 2009	401	54.2	various	CD38 [2,68], u*IGHV* [NR], telomere length [2.14], lymphocytosis [3.27], B2M [2.53]	NA	Telomere length [1.91], age [4.02], stage [2.14], B2M [NR]
102	Haferlach, Genes Chromosomes Cancer 2010	399	32.8	various	m*IGHV* [0.21], lymphocytosis [1.78],	NA	*TP53* [5.19], age [1.08], lymphocytosis [4.10]
2	Hallek, Lancet 2010	817	96	FC vs FCR	NA	u*IGHV* [1.51], 17p- [7.49], lymphocytosis [1.41], B2M [1,40]	17p- [9.32], PS [1.85], B2M [1.82], TK [1.87]
81	Oscier, Haematologica 2010	777	68	Chlor vs Fluda vs FC	NA	NA	NA
41	Rossi, AJH 2010	128	81	various	CD49d [2.10], u*IGHV* [2.01], telomere length [1.92], stage [3,69], lymphocytosis [2.19]	NA	NA
57	Zenz, JCO 2010	375	52.8	F vs FC	NA	*TP53* [3.78], 11q- [1.74]	u*IGHV* [1.92], 17p- [2.31], *TP53* [7.24], 11q- [1.91]
36	Gonzalez, JCO 2011	529	77	Chl vs F vs FC	NA	u*IGHV* [1.85], 17p- [3,28], *TP53* [1.77], 11q- [1.65], male sex [NR]	u*IGHV* [1.99], 17p- [5.75], 11q- [1.33], age [1.07]
90	Rossi, Blood 2011	301	-	various	NA	NA	*TP53* [3.14], *SF3B1* [3.02], age [3.17], stage [3.33]
70	Wierda, JCO 2011	930	26	various	u*IGHV* [10.68], 17p- [2.12], 11q- [1.86]	NA	NA
82	Bulian, J Transl Med 2012	620	120	various	NA	NA	u*IGHV* [2.04], 17p- [2.06], B2M [1.59], age [3.43], male sex [1.8], stage [3.68]
89	Rossi, Blood 2012	309	72	various	NA	NA	17p-*/TP53* [3.27], *NOTCH1* [3.99], stage [2.33], male sex [1.96], age [1.07]
33	Pepper, BJH 2012	1154	96	various	CD38 [1.60], u*IGHV* [3.30], age [NR]	NA	CD38 [1.70], u*IGHV* [2.70], age [NR]
62	Mansouri, AJH 2013	265	83	various	CD38 [1.92], 11q- [2.14], telomere length [1.93]	NA	CD38 [1.65], telomere length [2.42], age [2.04], stage [2.92]
54	Oscier, Blood 2013	494	120	Chl vs F vs FC	NA	u*IGHV* [1.86], *TP53* [2.09], 11q- [1.61]	u*IGHV* [1.85], *TP53* [2.48], 11q- [1.39], *NOTCH1* [1.58], *SF3B1* [1.52], age [1.05], male sex [1.39], stage [1.45]
71	Baliakas, AJH 2014	1001	NR	various	u*IGHV* [0.175], complex karyotype [0.435], stage [3.422]	NA	NA
37	Bulian, JCO 2014	2972	36	various	ZAP70 [1,46], CD49d [1.68], u*IGHV* [1.73], 11q- [1.48], 17p- [1.6], B2M [1.65], lymphocytosis [2.24]	NA	CD49d [2.26, u*IGHV* [2.48], 17p- [2.13], age [3.25], male sex [1.83], lymphocytosis [1.66]
42	Jeromin, Leukemia 2014	1160	55.2	various	u*IGHV* [3.03], 11q- [1.50], *SF3B1* [1.49]	NA	u*IGHV* [2.17], *TP53* [2.21], *SF3B1* [2.11], male sex [1.89]
43	Lin, BJH 2014	321	67.2	various	NA	u*IGHV* [1.21], telomere length [5.11], stage [1.52]	CD38 [2.67], telomere length [12.86]
34	Pflug, Blood 2014	1948	63.4	F, FC,FCR	NA	NA	u*IGHV* [2.10], 17p- [6.0], 11q- [1.4], age [1.30], sex [1.30], PS [1.70], B2M [2.30], TK [2.10]
55	Stilgenbauer, Blood 2014	497/507	70	FC vs FCR	NA	u*IGHV* [1.72], 17p- [2.92], *TP53* [2.12], 11q- [1.55], *SF3B1* [1.35], TK [1.36]	u*IGHV* [2.06], 17p- [2.72], *TP53* [3.01], age [1.42], PS [1.62], B2M [1.47], TK [1,86]
88	Baliakas, Leukemia 2015	774	72	various	u*IGHV* [3.70], *TP53* [2.08], 11q- [1,42], *SF3B1* [1.64]	NA	NA
56	Strefford, Leukemia 2015	384	120	Chl vs F vs FC	NA	u*IGHV* [1.59], *TP53* [2.51], 11q- [1.46], telomere length [2.10], male sex [1.34]	u*IGHV* [2.08], *TP53* [2.11], telomere length [2.21]
95	Rigolin, Genes Chromosomes Cancer 2015	250	50	various	u*IGHV* [2.164], 17p-/*TP53*mut [4.528], stage [3.345]	NA	17p-/*TP53*mut [4.305]; complex karyotype [3.630], stage [1.646]

uIGHV: unmutated configuration of the IGHV gene; PS: performance status; B2M: beta-2-microglobulin; TK: thymidine kinase; NR: not reported, NA: not applicable.
